# A Night in the Mountains

**Published:** 1873-08

**Authors:** 


### A NIGHT IN THE MOUNTAINS.

The practice of medicine in the rural districts has its lights as well as its shadows.

When one is wearied with the humdrum of daily business in town; when one has smiled and smirked until the risible muscles are worn with fatigue almost to exhaustion; when the respiratory organs have become clogged and choked with street dust; when the tympanum aches under the constant din of complaints real and complaints imaginary—how delightful to draw the reins over a fleet and sturdy horse, and speed one's course, upon a smooth and firm roadway, off to the mountains! Such was our involuntary ejaculation on yesterday, as we heartily responded to the call of a distant professional brother for counsel; and away we whirled, solitary and alone, for a five hours' drive through the beautiful and ever-varying mountain scenery of upper Georgia. Now speeding along through a fertile valley, teeming with luxuriant crops, promising rich return for the farmers' arduous toil; now down into a verdant meadow and among the sleek, contented kine—suggestive of full, foaming pails of the newly-drawn milk, of a cool, shaded dairy just below the bubbling mountain spring, of neat prints of golden butter for the evening meal; and now our road winds over a mountain spur, and elevates us into a higher and purer atmosphere, and we tarry a moment to expand our lungs again and again as we inhale the exhilarating oxygen, and feast our eyes upon the glittering display of tinted rays which shoot athwart the western horizon, and sport with the fleecy clouds in fairy forms of beauty.

What precious hours of communion with Dame Nature in her wildwood home! How delghtful to shake off for a brief period the contact of man with all his weaknesses, his foibles, his deceits, his corruptions!

Arrived at our destination, just as the twilight had deepened into sable night, we found the evening board already spread with the substantialities of life in tempting profusion, to which we paid our hearty devotion: and, coming to the matter of business, we were introduced by Dr. F. to his patient.

She was a lady of perhaps thirty-eight, staggering under the combined weight of pulmonary tuberculosis, limited hepatization, following recent sub-acute pneumonia, broken down stomach, endo-metritis of some years duration, menorrhagia, hectic emaciation. Truly heavy load to carry.

What are we to do with such a case? There is every incentive to do *something*. This lady is the sole stay and comfort of her aged parents; the idol of her husband, the *mother* of her children. She is an ornament to society—she is a power in her church. Surely her life is worth the saving if it be possible. But how? Will calomel save her? Will quinine? Will morphine? Will iodine? Will the emplastrum cantharidis?

We freely confess we have no faith in the power of such medicaments, nor indeed of any medicaments to rescue such cases. Our hope lies alone in the "*vis medicatrix;*" but surely we will assist nature by our medicines, as far as we may be able, in *supporting the powers of life*. We will order for her the syrup iodide iron, because our experience has shown this to be a prompt means of rallying a lost appetite—we will order Dr. Squibb's preparation because we have found it to be incomparably the best to be found in the market. We will order highly nutritious diet, and, especially, freshly-laid eggs, and all the rich cream from the cool dairy that her stomach will tolerate; and we will have this weighted down with a little good brandy, to assist its digestion. We will make it a point to get her out into the pure mountain air as soon as is practicable.

If we can give her appetite and digestive power with our ferric iodide and our alcohol, then we can feed and nourish her—then we can put flesh upon her bones; and if we can put flesh upon her bones, and *enough of it*, she will need no quinine for her fever, no calomel for her biliousness, no morphine nor expectorants for her cough. If she continue to *starve,* all the quinine and calomel and morphine in christendom can't rescue her from an early grave.

Learning that the neighborhood nurse and midwife, Mrs. T., was seriously ailing, and needed additional help, Dr. F. accompanied us to her home, some three miles off, where we met her attending physician, Dr. S., in consultation. Mrs. T. had labored under an umbilical hernia for more than twenty years, which had always been easily reducible until two years ago, since which time the return of the intestinal convolutions through the abdominal ring has been entirely prevented by adhesions. For two days the sac has been strangulated, and there was the usual pain, constipation, nausea, and vomiting, which attend intestinal obstruction.

Doctors S. and F., had each assayed the taxis, but without avail, and they had applied to the hernial sac an ingenious combined splint and compress, consisting of an ordinary earthenware saucer enveloped in a long bandage, which surrounded the body in such manner as to embrace the hernia in the bowl of the saucer. Upon examination we found a hernial mass, globular in form, measuring about six inches in diameter, with an abdominal ring of say two and a half inches diameter.

On the previous day the coils of intestine were much distended, with scybalous masses of quite firm consistency; these were now very much softened down into a pulpy material which yielded readily under the touch; and Dr. S. having put the patient well under the influence of opium, she was having free evacuations of the bowels in response to repeated doses of pills and castor oil, which had been previously taken.

The case was already, in great degree relieved, and it was determined to continue the opiate and purgatives, and likewise the saucer, which seemed to give support and protection to the hernia and comfort to the patient.

We have more than once seen an apparently formidable obstruction of the bowels yield most happily when the patient has been placed well under the influence of opium, and taken a very large dose of pills or calomel.

While tarrying an hour that we might avail ourselvs of moonlight to return, we improved the time in social and professional chit-chat. Dr. S. related the particulars of a case in his care, across the mountain, as interesting as it is remarkable.

Mr. ——, aged sixty-five, had retention of urine. Called Dr. S., who made repeated, but unsuccessful attempts to catheterize him. Dr. B. was called in consultation, and likewise failed to pass the catheter. Various other expedients were tried, but all to no purpose. *It is now more than two months since he has voided any urine.* During the greater portion of this time there has been copious diaphoresis, and when the hand is placed upon his forehead and moistened with his perspiration, it has a strongly urinous odor, as has also his bedding, and the atmosphere of his apartment. There has been *positively* no escape of urine from the bladder during this long period, either by the urethra, rectum, or by fistula. His intellect is clear, his appetite is good, and he eats bread and meat heartily for an invalid, and yet he is emaciated to a living skeleton.

Dr. S. promises us the full particulars of this unique case in a contribution to the JOURNAL, which we shall look for with interest.

The recital of the above case recalled to the mind of Dr. F. the case of an infant which he had delivered last year, which presented an icteroid hue of the skin. It survived to six months age, and during the whole period had white evacuations from the bowels, which repeated doses of calomel and other purgatives failed to change at all in color. The child perspired very freely, and the clothing was habitually dyed of a greenish yellow color by the sweat. This was uniform, and continued throughout life. There was no post-mortem. Dr. F. is of opinion that there was probably congenital closure of the common biliary duct.

Rough as is the life of our mountain doctors, it is not wholly without its attractions. For the most part they are men of estimable qualities—men of simple, unaffected manners, of warm, genial hearts, and of a worth far beyond their outward seeming. Very many of them possess a strength of intellect and a culture which would in no sense disgrace our richly upholstered city drawing-rooms. With their scanty assortment of well-thumbed college text books, their weekly mail on horseback, bringing, at long intervals, a single medical periodical, we are often surprised to find them *au courant* with the latest time; whilst their fruitfulness in expedients to patch out the deficiencies in their armamentarium often elicits our warmest praise. The remote and obscure accoucheur who safely delivers a travailing woman with a rude pot-hook from the kitchen chimney, dexterously guarding its dangerous point with his finger, is surely not to be despised in any circle of professional society. B.

